# Intercalation of Glyphosate in Mg–Al Layered Double Hydroxides and Its Controlled Release

**DOI:** 10.1021/acsomega.5c10605

**Published:** 2026-01-30

**Authors:** Emanoel Hottes, Gladson de Souza Machado, Glauco Favilla Bauerfeldt, Rosane Nora Castro, Marcelo Hawrylak Herbst

**Affiliations:** Instituto de Química, 67825 Universidade Federal Rural do Rio de Janeiro, CEP 23890-000 Seropédica, RJ, Brasil

## Abstract

The LDHs in the Mg–Al systems containing glyphosate were synthesized by the reconstruction and coprecipitation methods at a constant pH. The XRD data show that the Mg_2_Al–glyphosate hybrid material was formed in both cases, even though a better crystallinity was achieved from the synthesis conducted by the coprecipitation method. Moreover, vertical glyphosate intercalation can be inferred, given the observed 7.9 Å interlamellar spacing. Solid-state NMR and FT-IR-ATR data corroborate the formation of the material, with significant changes in the spectra of both when comparing the hybrid materials and free glyphosate. Studies involving pH variation showed the greatest glyphosate release at pH 10, reaching approximately 70% and governed by first-order kinetics, confirmed by the simulation model. Experiments involving carbonate, nitrate, and chloride anions suggest that the presence of carbonate has a greater influence on glyphosate release and that an increase in the concentration of this anion also corroborates to an increase in glyphosate release, both of which are also governed by a first-order mechanism.

## Graphical abstract

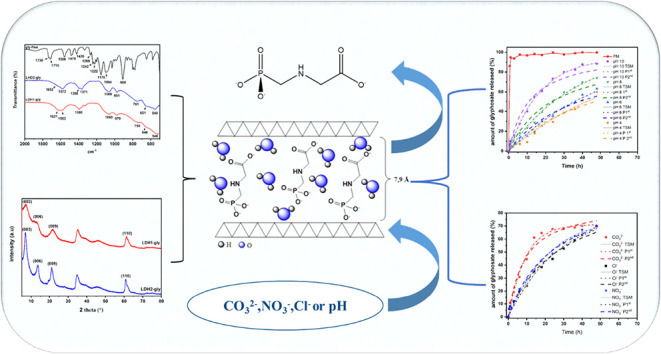

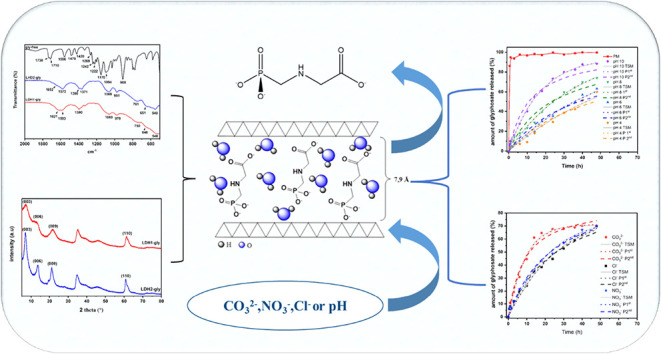

## 1 Introduction

N-(Phosphonomethyl)­glycine, commonly known as glyphosate, is a nonselective, systemic, postemergence herbicide with a broad-spectrum herbicide that is widely used in many countries, including Brazil,^1–3^ being applied to tobacco, soybean, sugarcane, rice, and other crops.^4–6^ Its principle of action is based on inhibiting the EPSPS (5-enol-pyruvyl-shikimate-3-phosphate synthase) enzyme, which is fundamental in the biosynthesis of amino acids.^7^ One of the major concerns associated with glyphosate lies in its degradation product, aminomethylphosphonic acid (AMPA), which has a half-life relatively longer than that of its precursor. In addition to biological degradation, glyphosate is susceptible to processes, such as leaching and percolation, which are influenced by weathering and soil composition. Upon reaching deeper layers, glyphosate may reach groundwater, causing contamination and subsequently affecting water bodies by rendering them unsuitable due to the loss of minimum potability standards.^8–13^ Current studies reveal that chronic exposure to glyphosate, from water and food, may be associated with a range of comorbidities, including attention-deficit/hyperactivity disorder (ADHD), colitis, diabetes, heart disease, intestinal inflammation, multiple sclerosis, obesity, depression, Alzheimer’s disease, autism, birth defects, brain and breast cancer, celiac disease and gluten intolerance, chronic kidney disease, and allergies, among others.^14–20^ An alternative in the search for mitigating the harmful effects of glyphosate may be through its application through slow-release systems, in which this active ingredient would be made available gradually, preventing it from being rapidly transformed into degradation byproducts and drained into the inner layers of the soil. For this purpose, layered double hydroxides (LDHs) are potentially interesting materials, given the versatility of such compounds.^21^ LDHs are anionic clay minerals with a brucite-type chemical structure, where a divalent metal is isomorphically replaced by a trivalent metal, leading to the formation of a positive charge density, which is stabilized by organic or inorganic anions, as well as water molecules.^22,23^ The chemical formula for LDHs is represented by [M_1–*x*_^2+^ M_*x*_^3+^ (OH)_2_]^*x*+^(A^*n*–^)_*x*/*n*_·mH_2_O, where M^2+^ and M^3+^ correspond, respectively, to divalent and trivalent cations, and A^n‑^ represents the anion present in the interlayer region. The ratio between the cations can vary from 1 to 6, corresponding to a range *x* of 0.15 ≤ *x* ≤ 0.5, where *x* = (M^3+^/M^2+^+M^3+^).^24^ The ratio between M^2+^ and M^3+^ cations will determine the layer charge density and, consequently, influence properties such as crystallinity, the amount of anions present in the interlayer domain, and the anion exchange capacity.^25–28^ In general, the possibility of variations in the composition of LDHs means that this material has a wide range of applications, including slow-release systems, flame retardants, environmental contaminant adsorbents, and catalysts.^24,29^ The application of LDHs that has also been gaining same attention is in the study of dye photodegradation, demonstrating the great versatility of these materials.^30,31^ However, its capacity as a slow-release material has attracted considerable attention. Studies involving the release of atrazine using the Mg_2_Al LDH over a period of 60 min showed a more intense release of the herbicide, around 33%, in the first 10 min, gradually decreasing until 60 min.^32^ The slow release of 2,4-D was investigated in a Zn–Al LDH system in the presence of solutions containing different anions to investigate their influence on the process. The phosphate anion showed the greatest effect on release, making it faster.^33^ The intercalation of the herbicide glyphosate from hybrid materials with LDHs is still not well explored in the literature. Studies involving the slow release of glyphosate intercalated in LDHs in the Zn–Al system indicated that after 48 h of contact with the aqueous medium, 70% of the glyphosate contained in the material was released into the medium, after which the system reached equilibrium.^21^ A study involving the Mg–Al–glyphosate hybrid material has been previously reported, but the effects of the concentration, pH, and synthetic routes were not explored.^34^ In this context, studies aiming at a deeper understanding of hybrid systems containing glyphosate remain highly relevant, given its increasing use across various countries. Accordingly, the present study focused on evaluating two synthetic routes for obtaining the hybrid compound and investigating the release behavior of the herbicide in aqueous media. Particular attention was given to the effects of pH variation and the concentration of different anions as well as to the kinetic mechanisms governing glyphosate desorption under each condition. The experimental data were fitted to the TSM isotherm model and to pseudo-first-order and pseudo-second-order kinetic models. For the sake of clarity, the materials were labeled according to the synthesis method employed. The Mg_2_Al–glyphosate LDH synthesized via the direct method, i.e., coprecipitation at constant pH, was designated as LDH1-gly. The material obtained through the reconstruction route, in which the carbonate-containing LDH was calcined and the resulting oxide was added to the glyphosate solution at pH 10, was designated as LDH2-gly. The Mg_2_Al–CO_3_ LDH prepared by coprecipitation at constant pH 10 and used as the precursor for the reconstruction of LDH2-gly was assigned the code LDH–CO_3_.

## 2 Materials and Methods

### 2.1 Materials

The reagents Na_2_MoO_4_, MgCl_2_·6H_2_O, AlCl_3_·6H_2_O, NaOH, Na_2_CO_3_, NaNO_3_, NaCl, HCl (36%), and ninhydrin (analytical grade), all sourced from Sigma-Aldrich, were used as received without the need for prior treatment. Glyphosate, on the other hand, was obtained from the commercial herbicide Roundup and processed according to the methodology described in the literature.^35^

### 2.2 Quantification of Residual Glyphosate in the Aqueous Solution and Glyphosate Content in the Hybrid Compound

Residual glyphosate present in the solutions after the release study was quantified by using a colorimetric method at 570 nm. The glyphosate content in the LDH was determined by opening the solid samples in an acidic medium, followed by derivatization and chromatographic analysis. Both the colorimetric assay and derivatization were performed following the protocol reported in the literature.^35^

### 2.3 Synthesis of the Layered Double Hydroxide Containing Glyphosate by the Reconstruction Method

LDH2-gly synthesized by reconstruction was obtained by adding 2 g of LDO to 250 mL of a solution containing 0.015 mol of glyphosate at pH 10. The suspension was left to react in a reactor for hydrothermal treatment (100 °C) for 72 h. After this step, the suspension was centrifuged, and the solid obtained was washed with water and ethanol and dried in a desiccator under vacuum conditions for 48 h. For comparison purposes, the LDH and LDO precursors, already properly characterized, are described in the literature and will be omitted here.^36^

### 2.4 Synthesis of the Layered Double Hydroxide Containing Glyphosate by the Direct Method

LDH1-gly obtained by the direct method was prepared according to the method described in the literature with some modifications.^34^ A total of 0.025 mol of MgCl_2_·6H_2_O and 0.0125 mol of AlCl_3_·6H_2_O was added to 100 mL of deionized water. This solution was slowly added dropwise (0.5 mL·min^–1^) to 250 mL of a solution containing 0.05 mol of glyphosate at pH 10. The pH of the medium was maintained by using a 0.1 M NaOH solution. The resulting suspension was then allowed to react under reflux (≈100 °C) for 24 h under an argon atmosphere. After this step, the solid was isolated following the procedure described in Section 2.3.

### 2.5 Study of Glyphosate Release in Aqueous Media as a Function of pH

All release assays as a function of pH were conducted in triplicate at room temperature. The residual glyphosate concentration was determined by using the colorimetric method described in Section 2.2. For comparison purposes, a release experiment was also performed using a physical mixture of the LDH and glyphosate. In this case, 37 mg of pure glyphosate and 113 mg of the LDH were mixed by trituration. The resulting mixture was then dispersed in 500 mL of distilled water at pH 6 and kept under static conditions for 48 h. Aliquots of 0.5 mL were withdrawn at predetermined time intervals (0.5, 1, 3, 6, 10, 14, 18, 24, 30, 36, and 48 h) for analysis. For the release studies from the LDH2-gly hybrid material, 150 mg of the solid was suspended in 500 mL of distilled water. Aliquots of 0.5 mL were collected at the same time intervals as described above. The glyphosate release from the hybrid material was investigated under different pH conditions (4, 6, 8, and 10). The pH of the medium during the release study was monitored using a pH meter, and 0.1 M HCl and 0.1 M NaOH solutions were used to keep it within the desired range throughout the experiment.

### 2.6 Study of Glyphosate Release in Aqueous Media as a Function of the Presence of Different Anions

The study of glyphosate release in an aqueous solution containing carbonate, nitrate, or chloride anions, all at a concentration of 5 × 10^–3^ M, was performed by adding 150 mg of the LDH2-gly hybrid compound to 500 mL of the respective solutions. To verify the glyphosate release rate, aliquots were collected from time to time as previously described. Due to the greater influence of the carbonate ion on glyphosate release, an investigation of the effect of different concentrations of this ion on herbicide release was performed. To this end, the release experiments were repeated using solutions containing concentrations of 2.5 and 10 × 10^–3^ M, in addition to the one described above.

### 2.7 Kinetic Models Adjusted to Release Studies

The desorption kinetics of glyphosate was investigated by applying a two-step mechanism (eq 1), which considers the elementary stages of adsorption and desorption as well as the pseudo-first-order (eq 2) and pseudo-second-order (eq 3) models.

1 $$\frac{d\theta }{dt}=k_{\mathrm{ad}}\cdot C\cdot \left(1-\theta \right)-k_{\mathrm{des}}\theta =k_{\mathrm{ad}}\frac{q_{\max}\cdot m_{\mathrm{LDH}}}{V}{\left(1-\theta \right)}^{2}-k_{\mathrm{des}}\theta$$

2 $$\frac{dq}{dt}=k_{p1}\left(q_{\mathrm{eq}}-q\right)$$

3 $$\frac{dq}{dt}=-k_{p2}{\left(q_{eq}-q\right)}^{2}$$

Since the two-step mechanism (eq 1) does not have an analytical solution, the ordinary differential equation was integrated using the third-order explicit Bogacki–Shampine method with a variable step size. Hermite polynomial interpolation was used to obtain the fraction of occupied sites **(q)** at the experimental time points, thus allowing the calculation of the model error. The pseudo-first-order and pseudo-second-order models were integrated analytically, with the initial condition set as *q = q*_max_ at *t = 0*. It is worth noting that for the pseudo-second-order model, it was necessary to assign a negative sign to the equation, as the desorption phenomenon occurs through a decrease in the fraction of occupied sites over time; therefore, its derivative must always be negative and tend toward zero as time approaches infinity. The model parameters *k*_ad_, *k*_des_, *k*_p1_, *q*_eq‑p1_, *k*_p2_, and *q*_eq‑p2_ were optimized using the sequential quadratic programming method by minimizing the sum of squared error between the experimental and modeled data. The parameters *k*_ad_ and *k*_des_ represent the rate coefficients of elementary steps of adsorption and desorption, respectively; *k*_p1_ and *k*_p2_ are the rate coefficients of pseudo-first-order and pseudo-second-order models, respectively; and *q*_eq‑p1_ and *q*_eq‑p2_ are the amounts of adsorbed glyphosate at equilibrium for the pseudo-first-order and pseudo-second-order models, respectively. The processes of numerical integration, interpolation, and optimization were carried out using algorithms already implemented in Octave 10.2.0 software.

## 3 Characterization

The solutions obtained from the colorimetric assay employed for the quantification of glyphosate in an aqueous solution were analyzed by using a Shimadzu UV–vis 1800 spectrophotometer. FT-IR/ATR spectra were recorded using a Bruker Vertex 70 spectrometer in the range of 400–4000 cm^–1^, with 128 scans performed at room temperature. Solid-state ^13^C­{^1^H} and ^31^P­{^1^H} MAS NMR spectra were acquired at 400 MHz using a Bruker Avance II spectrometer (9.4 T, standard probe, 4 mm ZrO_2_ rotors), operating at Larmor frequencies of 100 MHz for ^13^C and 168 MHz for ^31^P, respectively. The Mg/Al molar ratio of the precursor LDH was determined by metal quantification using an Agilent SpectrAA 55B atomic absorption spectrometer. Powder X-ray diffraction (PXRD) patterns were collected in the 2θ range of 5 to 80°, with a resolution of 0.2°, using a Rigaku Ultima IV diffractometer equipped with Cu Kα radiation (λ = 0.154 nm). The glyphosate content in the LDH matrix was determined using a Prominence-Shimadzu HPLC system comprising an LC-20AT pump, an SPD-M20A diode array detector, a CTO-20A column oven, a SIL-10AF autosampler, a CBM-20A system controller, and LabSolution software. Analyses were conducted on a C-18 Allure Organic Acids column (15 cm × 4.6 mm × 5 μm, Restek), under isocratic elution with a mobile phase composed of 80% acetonitrile (solvent B) and 20% phosphate buffer at pH 2.5 (solvent A), over a total run time of 4 min. The flow rate was set to 1.3 mL·min^–1^, the column temperature was maintained at 40 °C, and the injection volume was 20 μL. Detection of the herbicide was performed at 260 nm. Thermogravimetric analysis (TGA–DTG) was carried out in locally produced synthetic air by using a TA Instruments PCT-1A thermal analysis system. In this analysis, 21 mg samples were heated at a 5 °C min^–1^ rate from room temperature to 1000 °C.

## 4 Results and Discussion

### 4.1 Characterization of Materials

The hybrid compounds were obtained using a direct coprecipitation method at constant pH and by the reconstruction method, both at pH 10. For comparison purposes between the employed methods, the material synthesized by the classic coprecipitation method underwent conventional reflux heat treatment. The compound synthesized by the reconstruction method underwent hydrothermal treatment. It is worth noticing that intercalation, using the direct method, was only verified when excess glyphosate was used in the reaction medium, changing the gli/Al^3+^ ratio from 2:1, as formerly reported,^21,34^ to 4:1. Table 1 shows the percentage values of divalent and trivalent cations obtained in the synthesis of the materials as well as the average percentage of glyphosate.

**Table 1:** Percentage of Metals and Glyphosate Present in Solids Synthesized by the Direct Method and by Reconstruction

**sample**	**% Mg** ^**2+**^	**% Al** ^**3+**^	**Mg** ^**2+**^ **/Al** ^**3+**^	**% glyphosate**
LDH1-gly	16.61	7.76	2.14	20.95
LDH2-gly	17.01	8.54	1.99	24.53

Atomic absorption analysis confirmed that the experimental ratios of the di- and trivalent cations were consistent with the nominal values, i.e., those calculated and employed during the synthesis procedures. High-performance liquid chromatography with diode array detection (HPLC-DAD) indicated that the glyphosate content in the materials ranged from 20 to 24.5%. Analysis of the X-ray diffraction patterns, as shown in Figure 1, revealed that the synthesis via the reconstruction method followed by hydrothermal treatment yielded a material with a higher degree of structural order compared to that obtained through the coprecipitation method followed by reflux.

**Figure 1:**
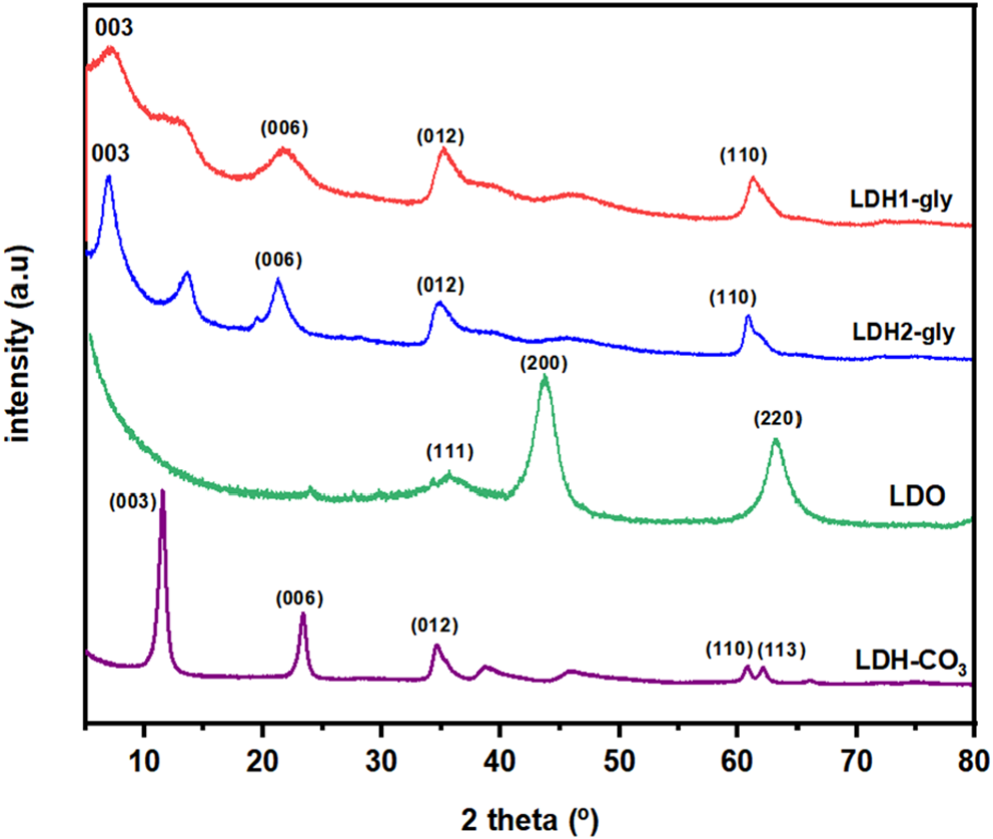
Comparison of diffraction patterns of glyphosate-containing LDHs (LDH1-gly and LDH2-gly) with calcined and uncalcined LDHs (LDO and LDH–CO_3_).

The ordering effect is evidenced by the XRD patterns, in which the diffraction peaks of the LDH2-gly sample appear to be sharper and better resolved. The basal spacing was calculated from the (003) reflection, yielding 12.6 and 12.7 Å for LDH1-gly and LDH2-gly, respectively. The interlayer spacing was estimated by subtracting the average thickness of the brucite-like layer (∼4.8 Å) from the basal spacing, resulting in interlayer distances of 7.8 and 7.9 Å. These values are in good agreement with those reported in the literature for structurally analogous layered double hydroxides intercalated with glyphosate. It was also possible to infer the presence of glyphosate in the interlayer region based on the XRD pattern obtained for the Mg–Al precursor solid prepared by the reconstruction method. The typical basal spacing value of 7.85 Å for carbonate-intercalated LDHs is lower than that observed for the basal spacing of the materials containing glyphosate; upon examining the diffractogram of the calcined material, it is possible to observe the collapse of the LDH layer structure, as evidenced by the disappearance of the (001) diffraction peaks. The diffractogram is typical of low-crystallinity mixed oxides with an MgO-type structure, in which the presence of Al^3+^ cations causes a shift of the reflection lines relative to cubic-phase MgO.^34,37^ The interlayer spacing is directly influenced by the orientation of the intercalated anion, and for glyphosate, such an orientation depends on the anionic form in which it is present. For pH values ranging from 9 to 10, two anionic species are possible, gly^2–^ and gly^3–^, and the ratio between them will depend on how close the pH of the medium is to 9 or 10. For pH values greater than 10, the gly^3–^ species is predominant.^38^ The presence of multiple species in the medium may contribute to the variations in interlayer spacing reported in the literature for materials synthesized under similar conditions. Studies involving the synthesis of LDHs in the Zn–Al–glyphosate system at pH 9 reported a basal spacing of 9.1 Å, suggesting horizontally intercalated glyphosate.^21^ According to Meng and co-workers, in systems involving Mg–Al–glyphosate LDHs, intercalation occurs in a horizontal orientation at pH 10, with glyphosate in the gly^3–^ form, while vertical intercalation is observed at pH 9.^34^ An ion exchange study using the glyphosate solution at pH 10, proposed by Li and collaborators and employing the coprecipitation method for the Mg–Al–glyphosate system, obtained an average interlayer domain value of 7.4 Å, suggesting vertical intercalation.^37^ According to the authors, glyphosate may interact with the lamellae through its phosphonate and carboxylate groups via strong hydrogen bonds mediated by water molecules acting as bridges in addition to electrostatic interactions with the positively charged layers. The interlayer domain values of 7.8 and 7.9 Å obtained in this study, which are higher than those previously reported in the literature, suggest that vertically intercalated glyphosate forms an organic monolayer and possibly interacts with water molecules associated with the LDH layer as well as directly with the layer itself. Vertical intercalation was also proposed based on theoretical studies carried out by the group, in which glyphosate shows an estimated molecular size greater than 6 Å.^38^ Considering the similarity in the basal spacing and interlayer domain values obtained for both materials, Figure 2 represents the intercalation mode of glyphosate in both LDH1-gly and LDH2-gly.

**Figure 2:**
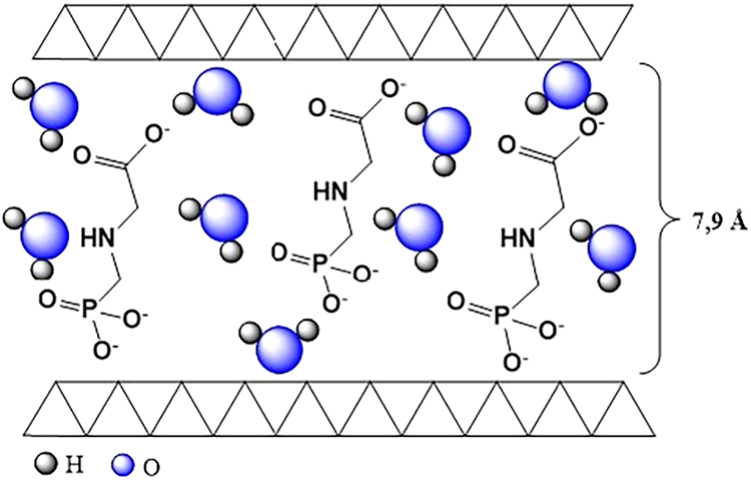
Representation of the interlayered spacing of hybrid materials LDH1-gly and LDH2-gly.

The FT-IR/ATR spectra for the LDH1-gly and LDH2-gly solids in the 2000–500 cm^–1^ region are shown in Figure 3, while the complete spectra are presented in Figure S1. From the analysis of the spectra, a broad band around 3300 cm^–1^ is observed for both materials, which is attributed to the vibrational modes of the −OH groups from the layer structure and from intercalated and/or surface-adsorbed water molecules.^36^ The intercalation process led to a shift in the main absorption bands when compared with those of free glyphosate. Changes were observed in the vibrational modes associated with both carboxylate and phosphonate groups. The band observed at 1710 cm^–1^, with a shoulder at 1730 cm^–1^, corresponding to the vibrational modes of the COO^–^ group of free glyphosate, was shifted to lower wavenumbers after intercalation, appearing as a broadened band with absorption maxima at 1573 cm^–1^ for LDH2-gly and 1583 cm^–1^ for the LDH1-gly solid.^36,37,39,40^

**Figure 3:**
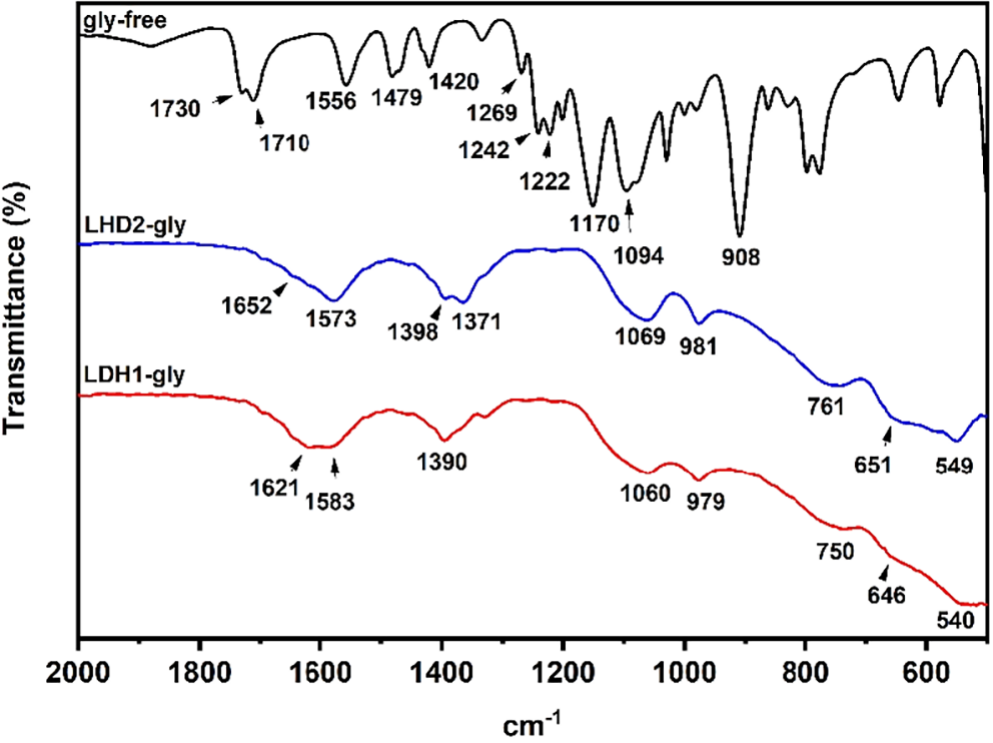
FT-IR/ATR spectra in the range of 2000–500 cm^–1^ of free glyphosate, the solid obtained by reconstruction (LDH2-gly), and the solid obtained by the direct method (LDH1-gly).

As previously reported, the C=O bond exhibits a complementary vibrational mode, observed at 1420 cm^–1^, after intercalation, which appears as a broad band with a maximum at 1398 cm^–1^ for LDH2-gly and 1390 cm^–1^ for LDH1-gly. Literature reports a similar shift in the region of 1400 cm^–1^.^34,37,39–41^ The vibrational modes corresponding to the P–O bonds in the (PO_3_^2–^) moiety, for free glyphosate, are usually recorded as two well-defined bands at 1170 and 1094 cm^–1^. After intercalation, these absorptions were observed as a broad band with maxima at 1060 and 1069 cm^–1^ for LDH1-gly and LDH2-gly, respectively.^34,37^ It was also observed that this shift occurs as a broad band in the 1125–1079 cm^–1^ region, and this broadening was attributed to band overlapping. Finally, the bands observed in the 800–500 cm^–1^ region are typical of the vibrational modes of the O–M–O and M–O–M bonds of the layer structure.^34,37^ Figures S2 and S3 show the ^31^P­{^1^H} CP-MAS and ^13^C­{^1^H} CP-MAS NMR spectra of the hybrid compounds LDH1-gly and LDH2-gly, along with their respective simulations obtained using DMFIT software, as well as the ^31^P spectrum of free glyphosate. As shown in the ^31^P spectrum (Figure S2), the sample of free glyphosate displays only one signal with a δ_iso_ of 18 ppm, corresponding to the main transition (+1/2 → −1/2). The ^31^P spectra of the LDH1-gly and LDH2-gly samples exhibit three chemical shift signals: a strong one at 23 ppm, a less intense signal at 14 ppm, and a shoulder at 9 ppm. According to the literature, the appearance of multiple signals indicates significant variations in the chemical environment of the ^31^P nucleus.^42,43^ Also, according to the literature, signals shifted to lower chemical shift values compared to free glyphosate (18 ppm) suggest the complexation of glyphosate with the metals present in the layer. This chemical shift reflects a renewed shielding effect through interactions, such as hydrogen bonding, between the phosphonate group and the layer. The presence of two signals (at 9 and 14 ppm) in the high-field region may indicate that the phosphonate moiety is complexed to the metal centers in the layer in either a monodentate or a bidentate coordination mode.^43,44^ Studies on the interaction of phosphate anions with alumina have shown the possibility of both monodentate and bidentate coordination to the metal in the layered.^43^ The shielding process may also be favored by hydrogen-bonding interactions possibly occurring between the phosphonate moiety and water molecules located between glyphosate and the layer, as shown in Figure 2, suggesting intercalation. It is also observed that the interaction of glyphosate with LDHs leads to the formation of deshielded ^31^P sites, as evidenced by the signal recorded at 23 ppm. Li and coauthors observed a similar variation in the chemical shift in their study on the thermal behavior of LDHs in the Mg–Al system containing intercalated glyphosate. According to the authors, this deshielding may result from electrostatic interactions between glyphosate and the layered structure.^37^ After intercalation, a downfield shift of the ^13^C signals (Figure S3) was observed for both LDH1-gly and LDH2-gly. The simulation based on the experimental spectrum indicated that the signal previously recorded at 169 ppm, attributed to carboxylic carbon, shifted downfield and split into three signals: a broad peak at 180 ppm and two partially overlapping signals with maxima at 172 and 171 ppm for both analyzed solids. This splitting indicates the presence of at least three distinct chemical environments for the carboxylic ^13^C nucleus. According to Li and co-workers, glyphosate may be associated with the layered structure through complexation since this anion exhibits a high coordination potential. Complexation may occur either through the phosphonate group or through the carboxylate group.^43^ Figure 4a,b presents the TGA-DTG profiles for the LDH1-gly and LDH2-gly materials as a function of temperature. In both cases, the TGA curves exhibit three distinct regions of mass loss, each corresponding to well-defined peaks in the DTG curves. The first set of peaks, occurring below 300 °C, is associated with a continuous and apparent mass loss within the range of approximately 50 to 210 °C. These events are attributed to the release of physically adsorbed water on the external surfaces of the LDH structure and the subsequent removal of interlayer water molecules. The latter is desorbed at relatively higher temperatures due to the denser packing and stronger confinement within the interlayer galleries. The second major thermal event, observed at approximately 330 °C for the material synthesized via the direct method and at 415 °C for the reconstructed sample, is ascribed to the dehydroxylation of the brucite-like layers and the decomposition of interlayer anions. These thermal behaviors are in good agreement with previously reported data in the literature.^37^

**Figure 4:**
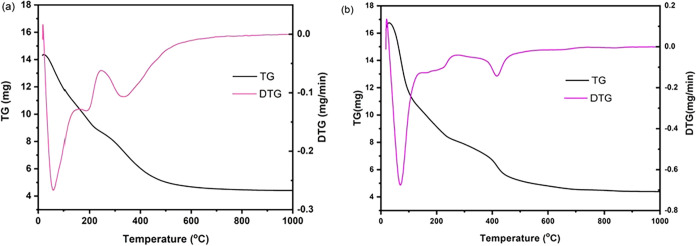
TGA thermograms of compounds (a) LDH1-gly and (b) LDH2-gly.

### 4.2 Evaluation of the Glyphosate Release Profile

Initially, to better understand the release behavior of glyphosate from the hybrid compound, a study was conducted in an aqueous medium under different pH conditions, varying from 4 to 10. Furthermore, the desorption of a physical mixture of LDH2-gly has also been carried out. Analysis of Figure 5 indicates that for the physical mixture, 100% of glyphosate present in the sample was released almost instantaneously compared to the hybrid material. This occurs because in the hybrid compound, glyphosate is bound to the LDH through electrostatic interactions, as well as hydrogen bonds. In contrast, in the material obtained from a physical mixture, glyphosate is not chemically bound and is therefore fully available for release. For simplicity, Figure 5 shows the release data for pH 4 and pH 10. The figure showing a comparison for all values can be seen in Supporting Figure 8.

**Figure 5:**
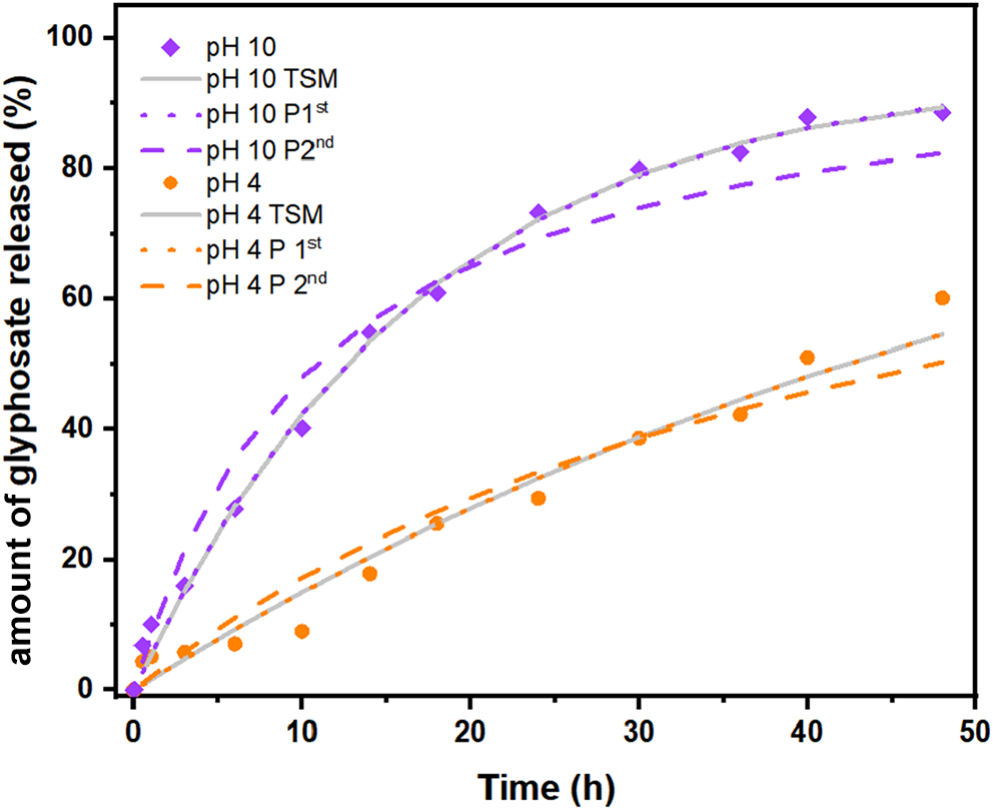
Glyphosate release in the aqueous medium from the hybrid compound LDH2-gly as a function of the pH value. Experimental values are represented by ■ (pH 10) and ● (pH 4). Continuous lines represent fittings according to the two-step kinetic mechanism (TSM), and dotted and dashed lines represent pseudo-first-order (P 1st) and pseudo-second-order (P 2nd) kinetic models, respectively.

By observation of the curves related to the glyphosate release in solution from the hybrid compound, it can be noted that the amount of glyphosate in solution increased with the contact time between LDH2-gly and water. The glyphosate release process is initially governed by the desorption step since under the initial condition, all of the sites are effectively occupied by the anion. However, as the fraction of occupied sites decreases due to the release, the concentration of glyphosate in solution increases, and as the experiment progresses, the adsorption and desorption rates become competitive until they equalize and the equilibrium is established. This is clearly observed at pH 10, where after 45 h of experiment, the curve begins to plateau, indicating that conditions close to equilibrium have been reached. As the pH increases, an increase in the desorption rate is observed. Considering a contact time of 10 h, glyphosate release increased from 10% at pH 4 to approximately 40% at pH 10, according to the experimental curve in Figure 5. A previously reported study shows an average glyphosate release rate of approximately 30% after 10 h of contact at pH 10, which is similar to the result obtained in the present work.^21^

A faster release at pH 4 would be expected, with respect to pH 10, as at lower pH, protonated glyphosate is bound less strongly between the LDH layers than at a higher pH. However, the construction of the LDH containing intercalated glyphosate is carried out in a way that ensures its anionic form for the purpose of stabilizing the layers. Once the material is synthesized, its internal structure does not change as a function of the external pH since glyphosate is already interacting with the LDH structure in different ways (electrostatic interactions and hydrogen bonding). In other words, the anionic form of intelayered glyphosate is unaltered by the external pH. The determining factor in the release process is the concentration of the external ion because the ion exchange mechanism will be predominant at this stage. Increasing the pH leads to a higher concentration of hydroxyl ions and, at even higher pH values, to the formation of carbonate in the medium due to the diffusion of atmospheric CO_2_, as the process was carried out under an ambient atmosphere.

For the case of pH 6, which is considered suitable for the development of various crops, it was observed that after 10 h of contact, approximately 15% of glyphosate present in the hybrid compound was released. After 24 h of contact time, the percentage of glyphosate released into solution increased from approximately 10 to 30% at pH 4 and from 40 to approximately 70% at pH 10. At pH 6, an average glyphosate release rate of more than 30% was observed within the same time period. Up to this point, it can still be noted that the desorption process remains dominant at all pH values. Through the fitting of experimental data to kinetic models, it was found that the process is governed by a first-order kinetic model attributed to the elementary desorption step. After 48 h of contact time, the glyphosate release rate was approximately 60% at pH values 4 and 6, reaching values close to 90% at pH 10, where, as previously mentioned, the onset of a plateau can be observed. The two-step kinetic mechanism (eq 1) considers the elementary stages of bimolecular adsorption and unimolecular desorption, assuming that a monolayer is formed and all sites are equivalent and independent, which is the same hypothesis of the TSM model, while the pseudo-first-order and pseudo-second-order kinetic mechanisms (eqs 2 and 3) evaluate the amount of adsorbed species as the system tends to the equilibrium condition. Through the analysis of Figure 5, an excellent agreement can be noted between experimental data and the fittings of the two-step kinetic mechanism and pseudo-first-order model; hence, it was found that the process is governed by a first-order kinetic model, attributed to the elementary unimolecular desorption step. Moreover, at pH 10, this agreement between the models is supported by the equilibrium constant values obtained for both, which were 159 and 181 L·mol^–1^ for the pseudo-first-order and two-step mechanism models, respectively, representing a deviation of only 12%. Additionally, as can be seen in Table 2, where the optimized kinetic parameters are shown for different pH experiments, the null value of *q*_eq‑p1_ obtained from the first-order model for experiments conducted at pH values lower than 10 further indicates that the system is still far from equilibrium and is being governed by the desorption step. For pH values lower than 10, the equilibrium is far from being reached, as can be seen in Figure 5; the adsorption rate coefficient (*k*_ad_) and the amount of glyphosate adsorbed at equilibrium (*q*_eq‑p1/2_) for pseudo kinetic model parameters were optimized to zero (Table 2); therefore, equilibrium constants could not be calculated for these experiments.

**Table 2:** Optimized Kinetic Parameters and Coefficient of Determination (*R* ^2^) for Different pH Values^a^

**parameters**	**p**H = 10	**p**H = 8	**p**H = 6	**p**H = 4
*k*_ad_ (M^–1^ h^–1^)	9.865874			
*K*_des_ (h^–1^)	0.054505	0.028138	0.019109	0.016092
*k*_p1_ (h^–1^)	0.059172	0.02814	0.019109	0.016092
*q*_eq‑p1_	0.00009174			
*k*_p2_ (g LDH mol gli^–1^ h^–1^)	60.9121	28.3028	17.24	13.8836

a Models: two-step mechanism (TSM) (*k*_ad_: rate coefficient of adsorption, *k*_des_: rate coefficient of desorption), pseudo-first-order model (*k*_p1_: rate coefficient, *q*_eq‑p1_: amount of glyphosate adsorbed at equilibrium), and pseudo-first-order model (*k*_p2_: rate coefficient, *q*_eq‑p2_: amount of glyphosate adsorbed at equilibrium).

The graph in Figure 6 presents the values of the adsorption and desorption rates at pH 10. It can be seen that at the initial stages of the experiment, these rates differ considerably but decrease over time. After 48 h, the adsorption rate is approximately half the value of the desorption rate, indicating that the system is approaching equilibrium. Phuong and collaborators obtained an average glyphosate release rate of approximately 70% over the same period of time, with the release curve in the aqueous medium indicating the onset of a stabilization trend in the process.^21^ Studies conducted at pH levels close to 7 involving different pesticides (picloram, 2,4-D, and MCPA) indicated a release process exceeding 90% after more than 48 h of contact between the hybrid compounds and the aqueous solution. In contrast, a study on the release of α-naphthaleneacetate attributed the process to several factors, such as the dissolution of the layer sheet, which may occur when the hybrid compound is exposed to more acidic solutions, ion exchange, an increased OH^–^ anion concentration, and the dissolution of atmospheric CO_2_ in experiments conducted under ambient atmospheric conditions.^33^

**Figure 6:**
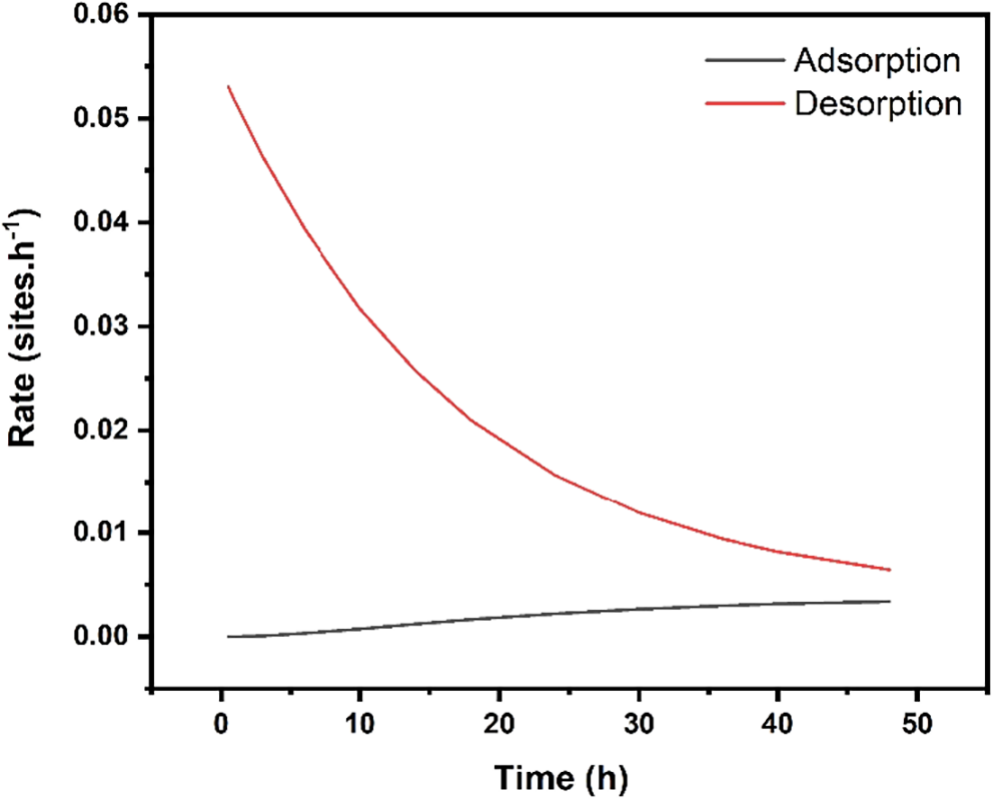
Adsorption and desorption rates for pH 10.

As previously mentioned, soil acidity is usually corrected by the addition of limestone. Liming has two main objectives: to reduce soil acidity and to supply calcium and magnesium to plants. As a consequence of the liming process, carbonate anions are present in the medium, which, in turn, may influence the release of glyphosate from the hybrid compound. In addition to the effect of carbonate anions, the influence of nitrate and chloride anions on the release process was also investigated, as shown in Figure 7. The ion exchange process, which is common in LDHs, intensifies desorption, causing the equilibrium between desorption and adsorption to be reached more quickly; i.e., it has a direct effect on the release kinetics. A higher release rate was observed when the experiment was conducted in a solution containing carbonate anions, followed by nitrate and then chloride. Within the first 10 h, a glyphosate release of 45% was observed. In the same period, the release of glyphosate at pH 6 and in the presence of chloride and nitrate anions was below 30%. After 15 h of contact, a release rate of 65% was observed, which remained constant at 70% after completing the 48 h cycle.

**Figure 7:**
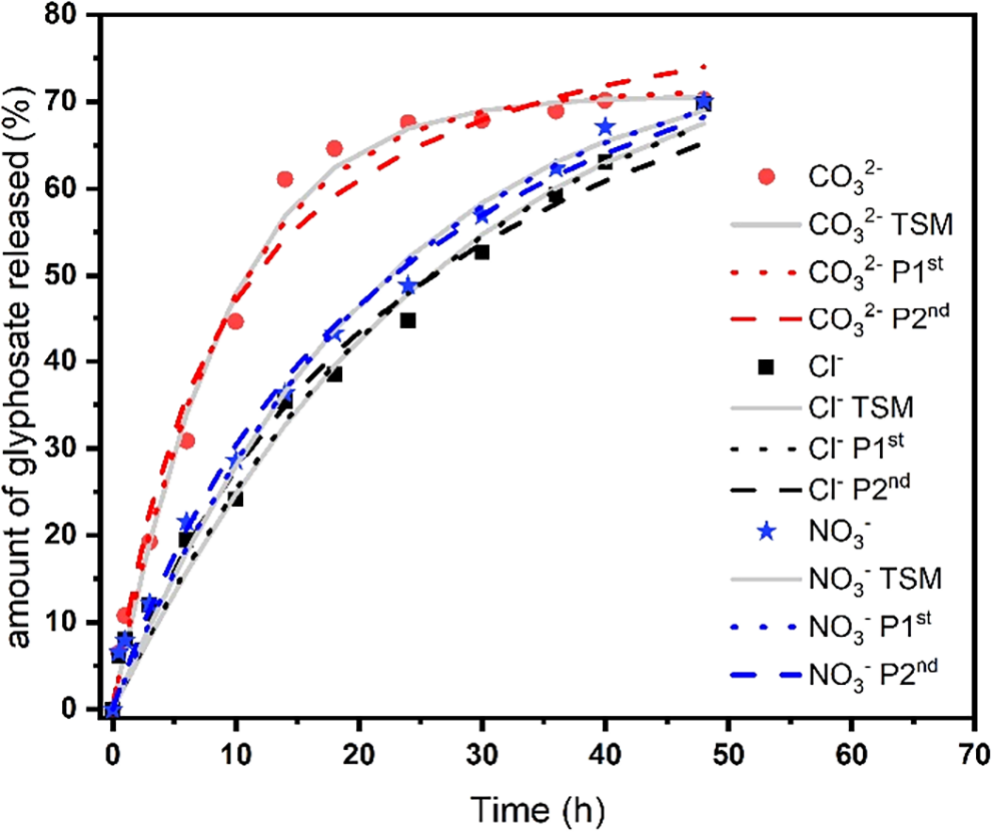
Release of glyphosate in the aqueous medium from the hybrid compound LDH2-gly as a function of different anions. Experimental values are represented by ● ([CO_3_^2–^] = 5 mM), ★ ([NO_3_^–^] = 5 mM), and ■ ([Cl^–^] = 5 mM). Continuous lines represent fittings according to the two-step kinetic mechanism (TSM), and dotted and dashed lines represent pseudo-first-order (P 1st) and pseudo-second-order (P 2nd) kinetic models, respectively.

The higher release rate provided by the carbonate anion is due to its greater capacity to stabilize the LDH, thus providing greater efficiency in the anion exchange capacity. According to Phuong and collaborators in their study on glyphosate release from Zn–Al LDH systems, carbonate was responsible for a higher release rate compared to chloride and hydroxide anions, reaching over 90% after 48 h. According to the authors, the higher release rate is due to the high anion exchange capacity of carbonate, which is associated with the synergistic effect attributed to its high charge and D_3h_ symmetry.^21^ In the present work, the release of glyphosate from the hybrid compound LDH2-gly in the presence of a chloride anion was higher than that reported in the literature. The OH^–^ anion has a greater stabilization capacity compared to the Cl^–^ anion; however, the concentration of Cl^–^ anions in solution at 5 mM is higher than the concentration of OH^–^ anions (Cl^–^ solutions have a pH ≈ 7), so it is possible to infer that this change in the release profile may be a result of the effect of this higher concentration. Although slower, the release gradually increases up to 48 h of contact time and maintains this growth profile. As reported in the pH study, the process follows first-order kinetics since it is governed by the desorption step. Hence, again, there was a good agreement between the two-step mechanism and the pseudo-first-order model, as can be seen in Figure 6 and Table 3, based on the comparison of the *R* ^2^ values and equilibrium constants.

**Table 3:** Optimized Kinetic Parameters and Coefficient of Determination (*R* ^2^) for Carbonate, Chloride, and Nitrate Anions at 5 mM^a^

**parameters**	**CO**_**3**_^**2–**^	**Cl** ^**–**^	**NO**_**3**_^**–**^
*k*_ad_ (M^–1^ h^–1^)	99.17	24.336	34.277
*K*_des_ (h^–1^)	0.070743	0.028321	0.032945
*R* ^2^ TSM	0.9929	0.9911	0.9941
*K*_eq_ TSM	1402	859	1040
*k*_p1_ (h^–1^)	0.11093	0.037654	0.045789
*q*_eq‑p1_	0.00043664	0.00030307	0.00034477
*R* ^2^ pseudo-1st	0.9915	0.9912	0.9945
*K*_eq_ pseudo-1st	1340	731	893
*k*_p2_ (g LDH mol gly^–1^ h^–1^)	93.232	25.4379	29.0312
*q*_eq‑p2_	0.00020889		
*R* ^2^ pseudo-2nd	0.9826	0.9865	0.9926
*K*_eq_ pseudo-2nd	432		

a Models: two-step mechanism (TSM) (*k*_ad_: rate coefficient of adsorption, *k*_des_: rate coefficient of desorption, *K*_eq_: equilibrium constant), pseudo-first-order model (*k*_p1_: rate coefficient, *q*_eq‑p1_: amount of glyphosate adsorbed at equilibrium, *K*_eq_ pseudo-1st: equilibrium constant), and pseudo-first-order model (*k*_p2_: rate coefficient, *q*_eq‑p2_: amount of glyphosate adsorbed at equilibrium, *K*_eq_ pseudo-2nd: equilibrium constant).

The success of the pseudo-first-order model compared to the pseudo-second-order model is due to the period during which the system is governed solely by the desorption step. To confirm this mechanism control hypothesis, an analysis of the rates of the adsorption and desorption steps was carried out based on the values of the fraction of occupied sites obtained from the numerical integration of the two-step mechanism with the optimized rate coefficients. As can be seen in Tables S1 and S2 of the Supporting Information, the rate of the desorption step was consistently considerably higher. Moreover, since in some experiments, the optimized rate coefficient for the adsorption step was equal to zero, as can be seen in Table 3, to ensure that this result was not a numerical instability, a sensitivity analysis of the fraction of occupied sites was performed for the experiment in the presence of 5 mM carbonate at the time instant of 48 h. This type of analysis aims to evaluate how errors due to uncertainty in the determination of a rate coefficient can be propagated in a kinetic mechanism.^45^ The greater the normalized sensitivity coefficient (in magnitude), the larger is the propagated error. The values of the normalized sensitivity coefficient for the adsorption and desorption steps were 0.52 and 0.56, respectively, the latter in absolute value; i.e., since these values are very similar, they propagate error similarly, and the mechanism is practically equally sensitive to both. Thus, it is demonstrated that in the experiments where the optimized rate coefficient for the adsorption step was zero, this is not a numerical instability but rather that this step did not have sufficient relevance to be determined. Due to the carbonate anion being responsible for a greater influence on the glyphosate release rate, the process was studied comparatively at three different concentrations: 5 mM, already presented previously, 2.5 mM, and 10 mM. The analysis of the graph shown in Figure 8 indicates that the glyphosate release rate is dependent on the carbonate anion concentration in the medium.

**Figure 8:**
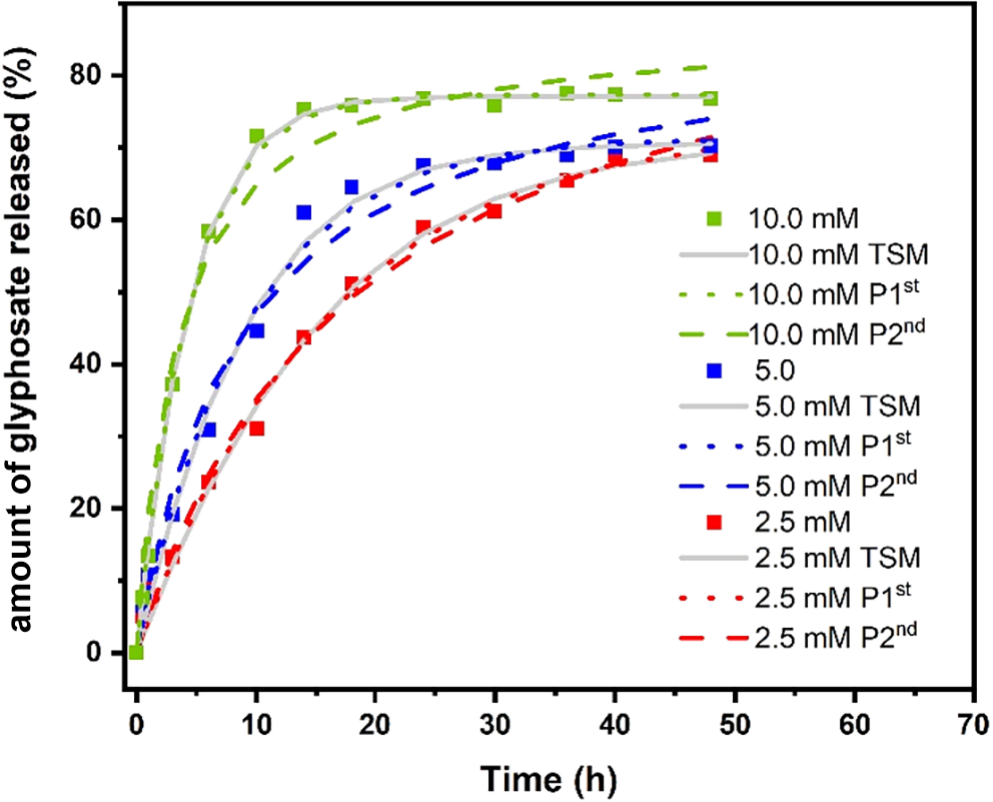
Release of glyphosate in the aqueous medium from the hybrid compound LDH2-gly as a function of the carbonate concentration. Experimental values are represented by ■ ([CO_3_^2–^] = 10 mM), ⧫ ([CO_3_^2–^] = 5 mM), and ● ([CO_3_^2–^] = 2,5 mM). Continuous lines represent fittings according to the two-step kinetic mechanism (TSM), and dotted and dashed lines represent pseudo-first-order (P 1st) and pseudo-second-order (P 2nd) kinetic models, respectively.

It can be observed that for the highest concentration, i.e., 10 mM, the release reaches its maximum at around 10 h (approximately 75%), remaining constant until the end of the 48 h cycle. For the more diluted carbonate solutions, glyphosate released remains constant after a contact time of approximately 20 h for the 5 mM carbonate solution and after 40 h for the 2.5 mM solution. Researchers observed in their study that the glyphosate release rate as a function of the carbonate concentration in the medium also increased with an increasing carbonate concentration, reaching 90% release after a 48 h contact period.^21^ However, the data observed in the present study agree with those obtained by Meng and collaborators, who observed that the release rate of glyphosate in a 6.5 mM carbonate solution increased up to an average contact time of 10 to 15 h, with no further increase in release after that time. This factor was attributed to the encapsulation process of glyphosate anions present in the innermost parts of the interlayer region, caused by a deformation of the LDH edges, preventing the passage of the glyphosate anion.^34^

## 5 Conclusions

The more defined diffraction peaks observed for the solid obtained through the reconstruction method followed by hydrothermal treatment (LDH2-gly) suggest that this material exhibits a better structural organization compared to the hybrid compound LDH1-gly, which was synthesized via the coprecipitation method at constant pH followed by reflux (direct method). Analysis of the diffractograms further indicated that in both materials, glyphosate is vertically intercalated, as evidenced by the average value of 7.8 Å obtained for the interlayer domains. The shifts in the absorption bands observed in the FT-IR/ATR spectra of intercalated glyphosate suggest interactions with the layers through the phosphonate and carboxylate groups, as previously predicted for the adsorption mechanism. Furthermore, the chemical shifts observed in the ^13^C­{^1^H} CP-MAS and ^31^P­{^1^H} CP-MAS NMR spectra indicate that glyphosate may be intercalated while interacting with the layers through complexation, in addition to electrostatic and hydrogen-bonding interactions. The pH-dependent release study (pH 4, 6, 8, and 10) indicated that increasing pH leads to an increase in the glyphosate release rate due to the higher OH^–^ concentration in the medium. The glyphosate release study from the hybrid material LDH2-gly in the presence of solutions containing different anions showed that carbonate anions promote a higher release rate of the organic compound compared to chloride and nitrate anions. It was observed that the glyphosate release rate increases with the increasing carbonate anion concentration in the medium. However, this release rate does not exceed 90%, as observed in the pH variation study, because the presence of carbonate anions results in the encapsulation of the glyphosate anions located deeper within the layers, thus hindering their release. The fitting of experimental release curves to mathematical models indicated that the release process both in the pH variation study and in the study involving different anions and concentrations follows a pseudo-first-order mechanism, as demonstrated by the best *R* ^2^ values obtained for this model.

## Supplementary Material



## Notes

FT-IR/ATR spectra in the range of 4000–500 cm^–1^ of free glyphosate, the solid obtained by reconstruction (LDH2-gly), and the solid obtained by the direct method (LDH1-gly) (Figure S1); ^31^P­{^1^H} CP-MAS spectra for free glyphosate, the solid obtained by reconstruction (LDH2-gly), and the solid obtained by the direct method (LDH1-gly) (Figure S2); ^13^C­{^1^H} CP-MAS spectra for free glyphosate, the solid obtained by reconstruction (LDH2-gly), and the solid obtained by the direct method (LDH1-gly) (Figure S3); adsorption and desorption rates (sites/h) for experiments in the presence of carbonate anions at different concentrations (Table S1); adsorption and desorption rates (sites/h) for experiments in the presence of chloride and nitrate anions at a 5.0 mM concentration (Table S2); sensitivity analysis; influence of the adsorption rate coefficient on the fraction of occupied sites (Figure S4); NSC determination for the adsorption step (Figure S5); influence of the desorption rate coefficient on the fraction of occupied sites (Figure S6); NSC determination for the desorption step (Figure S7); and glyphosate release in the aqueous medium from the hybrid compound LDH2-gly as a function of the medium’s pH; experimental values are represented by ■(PM representing the physical mixing between glyphosate and the hybrid compound),⧫ (pH 10),▼ (pH 8),▲ (pH 6), and ● (pH 4); continuous lines represent fittings according to the two-step kinetic mechanism (TSM) and dotted and dashed lines represent pseudo-first-order (P first) and pseudo-second-order (P second) kinetic models, respectively (Figure S8) (PDF)

## References

[1] Benbrook C. M. (2016). Trends
in Glyphosate Herbicide Use in the United States and Globally. Environ. Sci. Eur..

[2] Marques J. G. D. C., Veríssimo K. J.
D. S., Fernandes B. S., Ferreira S. R. D. M., Montenegro S. M. G. L., Motteran F. (2021). Glyphosate: A Review
on the Current Environmental Impacts from a Brazilian Perspective. Bull. Environ. Contam. Toxicol..

[3] De
Araujo L. G., Zordan D. F., Celzard A., Fierro V. (2023). Glyphosate
Uses, Adverse Effects and Alternatives: Focus on the Current Scenario
in Brazil. Environ. Geochem. Health.

[4] Martinez D. A., Loening U. E., Graham M. C. (2018). Impacts of Glyphosate-Based Herbicides
on Disease Resistance and Health of Crops: A Review. Environ. Sci. Eur..

[5] Richmond M. E. (2018). Glyphosate:
A Review of Its Global Use, Environmental Impact, and Potential Health
Effects on Humans and Other Species. J. Environ.
Stud Sci..

[6] Rawat D., Bains A., Chawla P., Kaushik R., Yadav R., Kumar A., Sridhar K., Sharma M. (2023). Hazardous
Impacts of
Glyphosate on Human and Environment Health: Occurrence and Detection
in Food. Chemosphere.

[7] Chen J., Huang H., Zhang C., Wei S., Huang Z., Chen J., Wang X. (2015). Mutations and Amplification
of EPSPS
Gene Confer Resistance to Glyphosate in Goosegrass (Eleusine Indica). Planta.

[8] Vereecken H. (2005). Mobility and
Leaching of Glyphosate: A Review. Pest Manage.
Sci..

[9] Borggaard O. K., Gimsing A. L. (2008). Fate of Glyphosate in Soil and the Possibility of Leaching
to Ground and Surface Waters: A Review. Pest
Manage. Sci..

[10] Khoury G. A., Gehris T. C., Tribe L., Torres Sánchez R. M., Dos Santos Afonso M. (2010). Glyphosate Adsorption on Montmorillonite: An Experimental
and Theoretical Study of Surface Complexes. Appl. Clay Sci..

[11] Espinoza-Montero P. J., Vega-Verduga C., Alulema-Pullupaxi P., Fernández L., Paz J. L. (2020). Technologies Employed in the Treatment
of Water Contaminated
with Glyphosate: A Review. Molecules.

[12] Carretta L., Cardinali A., Onofri A., Masin R., Zanin G. (2021). Dynamics of
Glyphosate and Aminomethylphosphonic Acid in Soil Under Conventional
and Conservation Tillage. Int. J. Environ. Res..

[13] Chávez-Ortiz P., Tapia-Torres Y., Larsen J., García-Oliva F. (2022). Glyphosate-Based
Herbicides Alter Soil Carbon and Phosphorus Dynamics and Microbial
Activity. Appl. Soil Ecol..

[14] Mink P. J., Mandel J. S., Sceurman B. K., Lundin J. I. (2012). Epidemiologic Studies
of Glyphosate and Cancer: A Review. Regul. Toxicol.
Pharmacol..

[15] Samsel A., Seneff S. (2013). Glyphosate, Pathways to Modern Diseases
II: Celiac
Sprue and Gluten Intolerance. Interdiscip. Toxicol..

[16] Samsel A., Seneff S. (2015). Glyphosate, Pathways to Modern Diseases III: Manganese,
Neurological Diseases, and Associated Pathologies. Surg. Neurol. Int..

[17] Gunarathna S., Gunawardana B., Jayaweera M., Manatunge J., Zoysa K. (2018). Glyphosate and AMPA
of Agricultural Soil, Surface Water, Groundwater
and Sediments in Areas Prevalent with Chronic Kidney Disease of Unknown
Etiology, Sri Lanka. J. Environ. Sci. Health,
Part B.

[18] Barnett J. A., Gibson D. L. (2020). Separating the Empirical Wheat From
the Pseudoscientific
Chaff: A Critical Review of the Literature Surrounding Glyphosate,
Dysbiosis and Wheat-Sensitivity. Front. Microbiol..

[19] Peillex C., Pelletier M. (2020). The Impact
and Toxicity of Glyphosate and Glyphosate-Based
Herbicides on Health and Immunity. J. Immunotoxicol..

[20] Barnett J., Yu K. K., Mayilvaganan B. (2024). S4714 Small Bowel Angioedema Secondary
to ACE Inhibitor. Am. J. Gastroenterol..

[21] Phuong N. T. K., Ha H. N. N., Dieu N. T. P., Huy B. T. (2017). Herbicide/Zn-Al-Layered
Double Hydroxide Hybrid Composite: Synthesis and Slow/Controlled Release
Properties. Environ. Sci. Pollut. Res..

[22] Vaccari A. (1998). Preparation
and Catalytic Properties of Cationic and Anionic Clays. Catal. Today.

[23] Sotiles A. R., Wypych F. (2020). Synthesis and Topotactic
Exchange Reactions of New
Layered Double Hydroxides Intercalated with Ammonium/Sulfate. Solid State Sci..

[24] Chubar N., Gilmour R., Gerda V., Mičušík M., Omastova M., Heister K., Man P., Fraissard J., Zaitsev V. (2017). Layered Double Hydroxides as the
next Generation Inorganic
Anion Exchangers: Synthetic Methods versus Applicability. Adv. Colloid Interface Sci..

[25] Radha A. V., Vishnu Kamath P., Shivakumara C. (2005). Mechanism of the Anion Exchange Reactions
of the Layered Double Hydroxides (LDHs) of Ca and Mg with Al. Solid State Sci..

[26] Jijoe P. S., Yashas S. R., Shivaraju H. P. (2021). Fundamentals,
Synthesis, Characterization
and Environmental Applications of Layered Double Hydroxides: A Review. Environ. Chem. Lett..

[27] Kameliya J., Verma A., Dutta P., Arora C., Vyas S., Varma R. S. (2023). Layered Double Hydroxide
Materials: A Review on Their
Preparation, Characterization, and Applications. Inorganics.

[28] Farhan A., Khalid A., Maqsood N., Iftekhar S., Sharif H. M. A., Qi F., Sillanpää M., Asif M. B. (2024). Progress
in Layered Double Hydroxides (LDHs): Synthesis and Application in
Adsorption, Catalysis and Photoreduction. Sci.
Total Environ..

[29] Mishra G., Dash B., Pandey S. (2018). Layered Double
Hydroxides: A Brief
Review from Fundamentals to Application as Evolving Biomaterials. Appl. Clay Sci..

[30] Balayeva O. O., Azizov A. A., Muradov M. B., Alosmanov R. M., Israfilli T. Sh., Hashimova S. J., Gasimov E. K., Rzayev F. H., Sadigov N. M., Abdullayev M. I. (2024). Noncovalent Doping of Fullerene (C60)
into ZnAl–LDH/PVA Matrix and Photocatalytic Degradation of
Methylene Blue and Congo Red from Water. J.
Inorg. Organomet. Polym..

[31] Ma Y., Chen X., Li P., Balayeva O. O., Liang X., Zhang Y., Yin Y., Wang X. (2025). Hybrids of Layered
Double Hydroxides and Sodium Dodecyl Sulfate: Structural Effects on
the Controlled Release of Pretilachlor. ACS
Omega.

[32] Touloupakis E., Margelou A., Ghanotakis D. F. (2011). Intercalation
of the Herbicide Atrazine
in Layered Double Hydroxides for Controlled-release Applications. Pest Manage. Sci..

[33] Hussein M. Z., Jaafar A. M., Yahaya A. H., Zainal Z. (2009). The Effect of Single,
Binary and Ternary Anions of Chloride, Carbonate and Phosphate on
the Release of 2,4-Dichlorophenoxyacetate Intercalated into the Zn–Al-Layered
Double Hydroxide Nanohybrid. Nanoscale Res.
Lett..

[34] Meng J., Hui Z., Evans D. G., Xue D. (2005). Novellayered
Pesticide Slow/Controlled
Release Materials-Supramolecular Structure and Slow Release Property
of Glyphosate Intercalated Layered Double Hydroxides. Chin. Sci. Bull..

[35] Hottes E., Bauerfeldt G. F., Herbst M. H., Castro R. N., San Gil R. A. D. S. (2021). Rapid
quantification of residual glyphosate in water treated with layered
double hydroxides using liquid chromatography. Braz. J. Dev..

[36] Hottes E., Da Silva C. O., Bauerfeldt G. F., Castro R. N., De Lima J. H. C., Camargo L. P., Dall’Antonia L.
H., Herbst M. H. (2022). Efficient
Removal of Glyphosate from Aqueous Solutions by Adsorption on Mg–Al-Layered
Double Oxides: Thermodynamic, Kinetic, and Mechanistic Investigation. Environ. Sci. Pollut. Res..

[37] Li F., Zhang L., Evans D. G., Forano C., Duan X. (2004). Structure
and Thermal Evolution of Mg–Al Layered Double Hydroxide Containing
Interlayer Organic Glyphosate Anions. Thermochim.
Acta.

[38] Peixoto M. M., Bauerfeldt G. F., Herbst M. H., Pereira M. S., Da Silva C. O. (2015). Study of
the Stepwise Deprotonation Reactions of Glyphosate and the Corresponding
p*K*
_a_ Values in Aqueous Solution. J. Phys. Chem. A.

[39] Piccolo A., Celano G. (1993). Modification of Infrared
Spectra of the Herbicide Glyphosate
Induced by pH Variation. J. Environ. Sci. Health,
Part B.

[40] Piccolo A., Celano G. (1994). Hydrogen-Bonding Interactions between
the Herbicide
Glyphosate and Water-Soluble Humic Substances. ET&C.

[41] Piccolo A., Celano G., Conte P. (1996). Adsorption of Glyphosate by Humic
Substances. J. Agric. Food Chem..

[42] Li F., Wang Y., Yang Q., Evans D. G., Forano C., Duan X. (2005). Study on Adsorption
of Glyphosate (N-Phosphonomethyl Glycine) Pesticide
on MgAl-Layered Double Hydroxides in Aqueous Solution. J. Hazard. Mater..

[43] Li W., Wang Y.-J., Zhu M., Fan T.-T., Zhou D.-M., Phillips B. L., Sparks D. L. (2013). Inhibition
Mechanisms of Zn Precipitation
on Aluminum Oxide by Glyphosate: A^31^ P NMR and Zn EXAFS
Study. Environ. Sci. Technol..

[44] Li W., Feng X., Yan Y., Sparks D. L., Phillips B. L. (2013). Solid-State
NMR Spectroscopic Study of Phosphate Sorption Mechanisms on Aluminum
(Hydr)­Oxides. Environ. Sci. Technol..

[45] Turányi T. (1990). Sensitivity
Analysis of Complex Kinetic Systems. Tools and Applications. J. Math. Chem..

