# Supplementary material for: Intercalation of Glyphosate in Mg–Al Layered Double Hydroxides and Its Controlled Release
Source: ACS Omega. 2026 Jan 30;11(6):9915–25. doi: 10.1021/acsomega.5c10605 (PMC12917639; doi:10.1021/acsomega.5c10605)

Supplementary material

Intercalation of glyphosate in Mg-Al layered double hydroxides and its controlled release

Emanoel Hottes[a], Gladson de Souza Machado[a], Glauco Favilla Bauerfeldt[a], Rosane Nora Castro[a] and Marcelo Hawrylak Herbst[a]*

[a]Instituto de Química, Universidade Federal Rural do Rio de Janeiro, CEP 23890-000 Seropédica, RJ, Brasil

*Email: hottes@ufrrj.br

Keywords: slow release, glyphosate, kinetic study, release dynamics

Figure S1: FT-IR/ATR spectra in the range of 4000 – 500 cm^{-1} of glyphosate free, solid obtained by reconstruction (LDH2-gly), and solid obtained by the direct method (LDH1-gly).

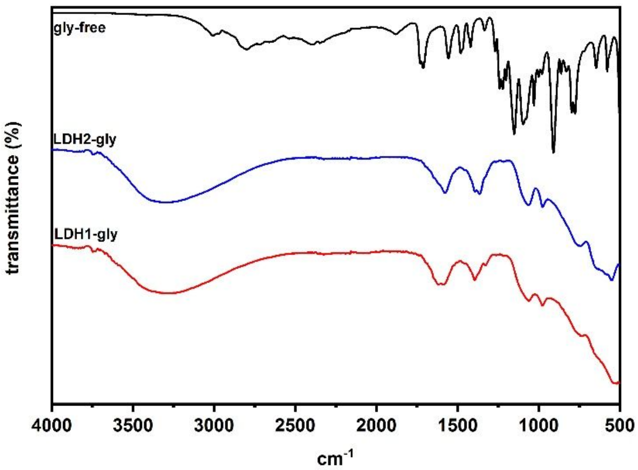

Figure S2: $^{31}P\{^{1}H\}$ CP-MAS spectra for glyphosate free, solid obtained by reconstruction (LDH2-gly), and solid obtained by the direct method (LDH1-gly).

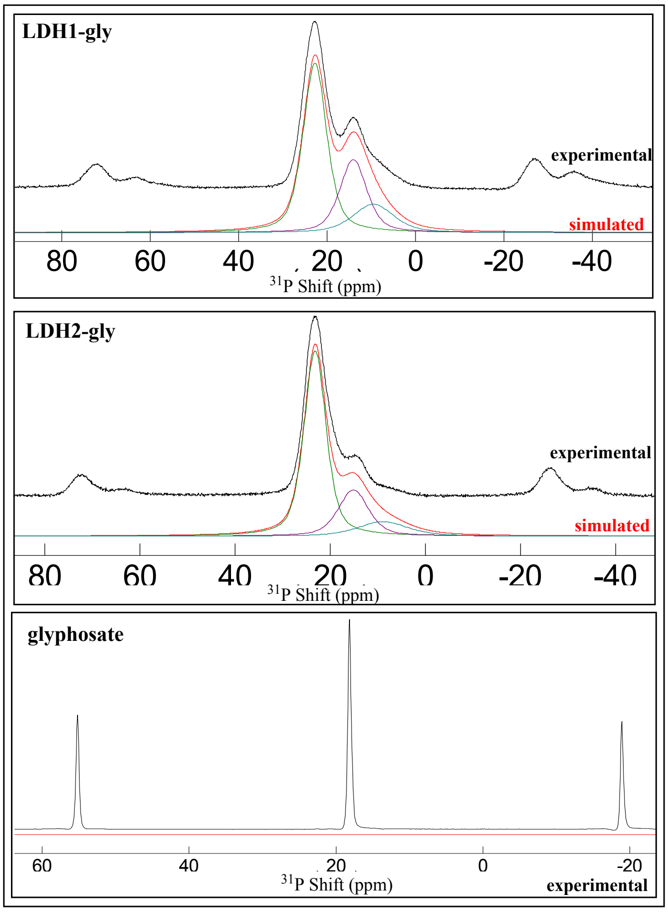

Figure S3: $^{13}C\{^{1}H\}$ CP-MAS spectra for glyphosate free, solid obtained by reconstruction (LDH2-gly), and solid obtained by the direct method (LDH1-gly).

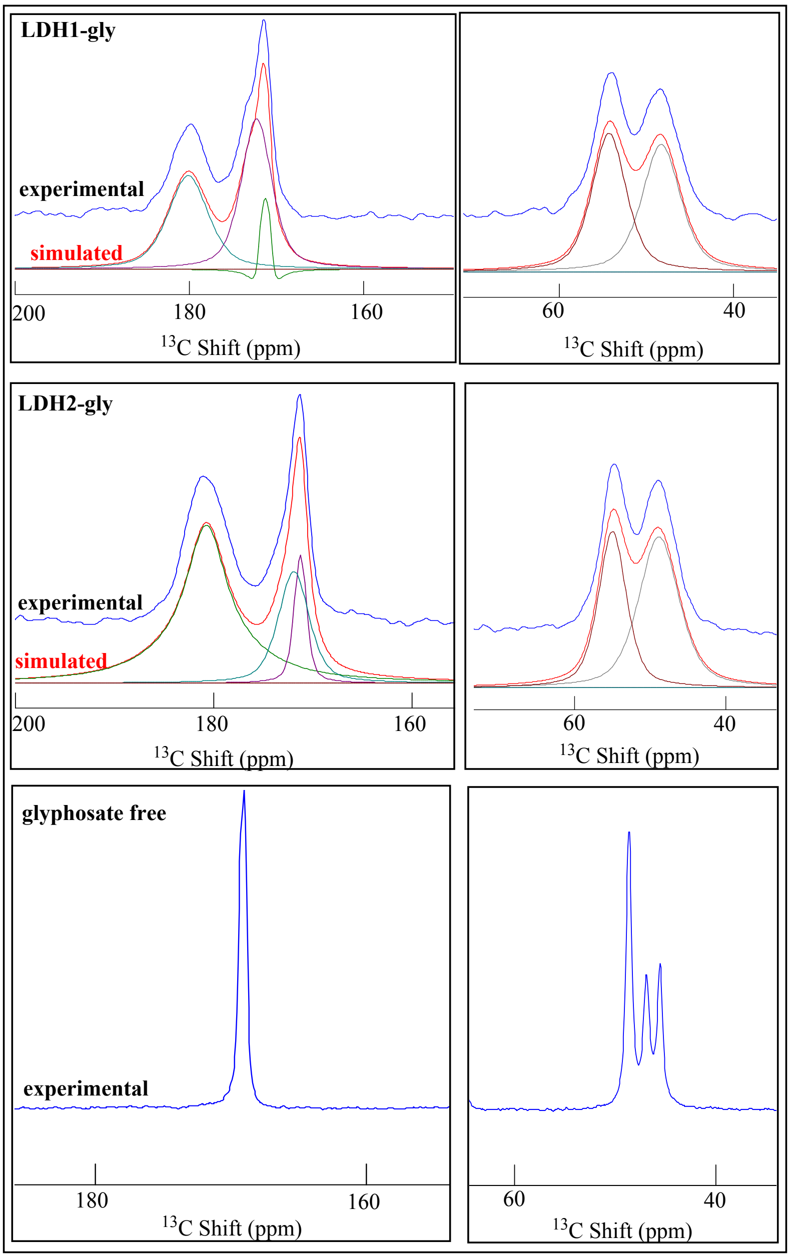

Table S1: Adsorption and desorption rates (sites/h) for experiments in the presence of carbonate anions at different concentrations.

	$[CO_3^{2-}]$ = 2.5 mM		$[CO_3^{2-}]$ = 5.0 mM		$[CO_3^{2-}]$ = 10.0 mM	
Time (h)	adsorption	desorption	adsorption	desorption	adsorption	desorption
0.5	$1.15x10^{-5}$	$4.20x10^{-2}$	$5.30x10^{-5}$	$6.83x10^{-2}$	$7.80x10^{-5}$	$1.13x10^{-1}$
1	$4.53x10^{-5}$	$4.11x10^{-2}$	$2.05x10^{-4}$	$6.59x10^{-2}$	$2.95x10^{-4}$	$1.07x10^{-1}$
3	$3.72x10^{-4}$	$3.77x10^{-2}$	$1.58x10^{-3}$	$5.73x10^{-2}$	$2.09x10^{-3}$	$8.41x10^{-2}$
6	$1.29x10^{-3}$	$3.33x10^{-2}$	$4.97x10^{-3}$	$4.70x10^{-2}$	$5.82x10^{-3}$	$5.98x10^{-2}$
10	$2.95x10^{-3}$	$2.84x10^{-2}$	$9.86x10^{-3}$	$3.73x10^{-2}$	$1.02x10^{-2}$	$4.01x10^{-2}$
14	$4.72x10^{-3}$	$2.45x10^{-2}$	$1.39x10^{-2}$	$3.10x10^{-2}$	$1.32x10^{-2}$	$2.93x10^{-2}$
18	$6.38x10^{-3}$	$2.15x10^{-2}$	$1.67x10^{-2}$	$2.72x10^{-2}$	$1.49x10^{-2}$	$2.35x10^{-2}$
24	$8.42x10^{-3}$	$1.83x10^{-2}$	$1.92x10^{-2}$	$2.40x10^{-2}$	$1.62x10^{-2}$	$1.95x10^{-2}$
30	$9.90x10^{-3}$	$1.63x10^{-2}$	$2.04x10^{-2}$	$2.26x10^{-2}$	$1.67x10^{-2}$	$1.80x10^{-2}$
36	$1.09x10^{-2}$	$1.49x10^{-2}$	$2.09x10^{-2}$	$2.19x10^{-2}$	$1.69x10^{-2}$	$1.74x10^{-2}$
40	$1.14x10^{-2}$	$1.43x10^{-2}$	$2.11x10^{-2}$	$2.17x10^{-2}$	$1.69x10^{-2}$	$1.72x10^{-2}$
48	$1.20x10^{-2}$	$1.36x10^{-2}$	$2.13x10^{-2}$	$2.15x10^{-2}$	$1.70x10^{-2}$	$1.70x10^{-2}$

Table S2: Adsorption and desorption rates (sites/h) for experiments in the presence of chloride and nitrate anions at 5.0 mM concentration.

	$[Cl^-]$ = 5.0 mM		$[NO_3^-]$ = 5.0 mM	
Time (h)	adsorption	desorption	adsorption	desorption
0.5	$2.13x10^{-6}$	$2.79x10^{-2}$	$4.06x10^{-6}$	$3.24x10^{-2}$
1	$8.42x10^{-6}$	$2.75x10^{-2}$	$1.60x10^{-5}$	$3.19x10^{-2}$
3	$7.15x10^{-5}$	$2.60x10^{-2}$	$1.34x10^{-4}$	$2.99x10^{-2}$
6	$2.62x10^{-4}$	$2.39x10^{-2}$	$4.84x10^{-4}$	$2.71x10^{-2}$
10	$6.45x10^{-4}$	$2.14x10^{-2}$	$1.16x10^{-3}$	$2.38x10^{-2}$
14	$1.12x10^{-3}$	$1.92x10^{-2}$	$1.97x10^{-3}$	$2.11x10^{-2}$
18	$1.64x10^{-3}$	$1.73x10^{-2}$	$2.82x10^{-3}$	$1.88x10^{-2}$
24	$2.42x10^{-3}$	$1.49x10^{-2}$	$4.01x10^{-3}$	$1.60x10^{-2}$
30	$3.15x10^{-3}$	$1.30x10^{-2}$	$5.05x10^{-3}$	$1.40x10^{-2}$
36	$3.80x10^{-3}$	$1.15x10^{-2}$	$5.89x10^{-3}$	$1.24x10^{-2}$
40	$4.17x10^{-3}$	$1.07x10^{-2}$	$6.35x10^{-3}$	$1.17x10^{-2}$
48	$4.79x10^{-3}$	$9.46x10^{-3}$	$7.05x10^{-3}$	$1.05x10^{-2}$

Sensitivity Analysis

The Normalized Sensitivity Coefficients (NSC) for the adsorption and desorption steps were calculated according to **Equation S1**. This calculation was performed using the data from the 5mM carbonate simulation at 48h time.

$$NSC = \frac{\partial \ln \theta}{\partial \ln k} \qquad \textbf{Equation S1}$$

To determine the NSC for the adsorption step, the desorption rate coefficient was held constant. Five simulations were performed varying the adsorption rate coefficient (0.5*kad, 0.75*kad, kad, 1.5*kad and 2.0*kad, where kad is the optimized rate coefficient). The results of these simulations are shown in **Figure S4**. Using the fraction of occupied sites at 48h and the values of rate coefficients, a natural logarithm plot was done. The NSC kad was determined as the slope of a lineat fit, as can be seen in **Figure S5**. The NSC for the desorption step was calculated by an analogous procedure, with results presented in Figures S6 and S7.

Figure S4: Influence of adsorption rate coefficient on the fraction of occupied sites.

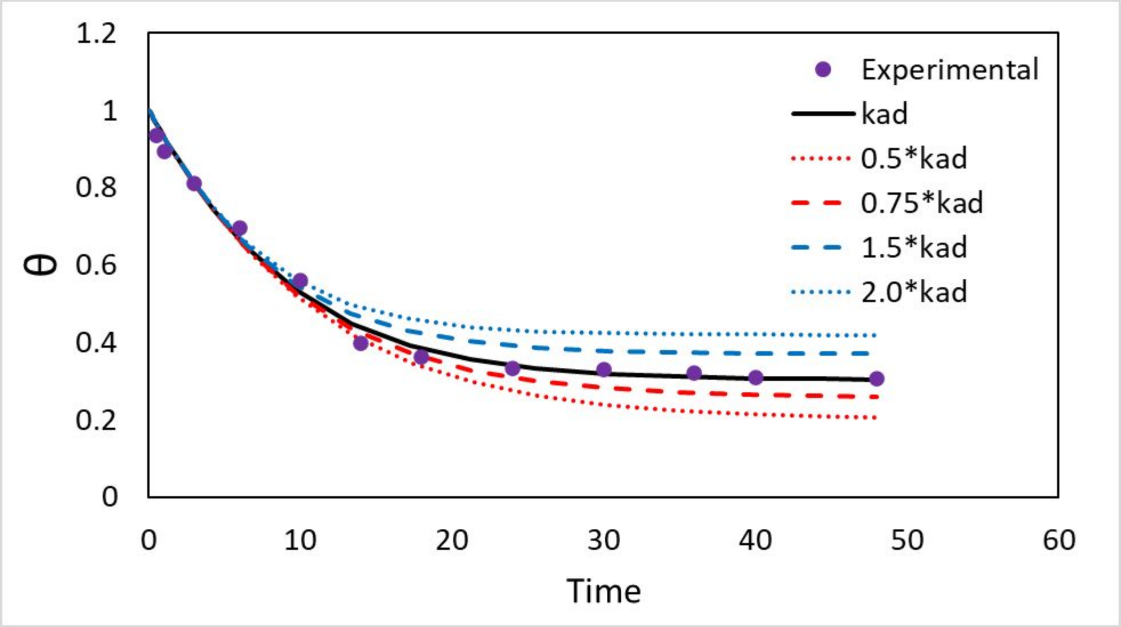

Figure S5: NSC determination for the adsorption step

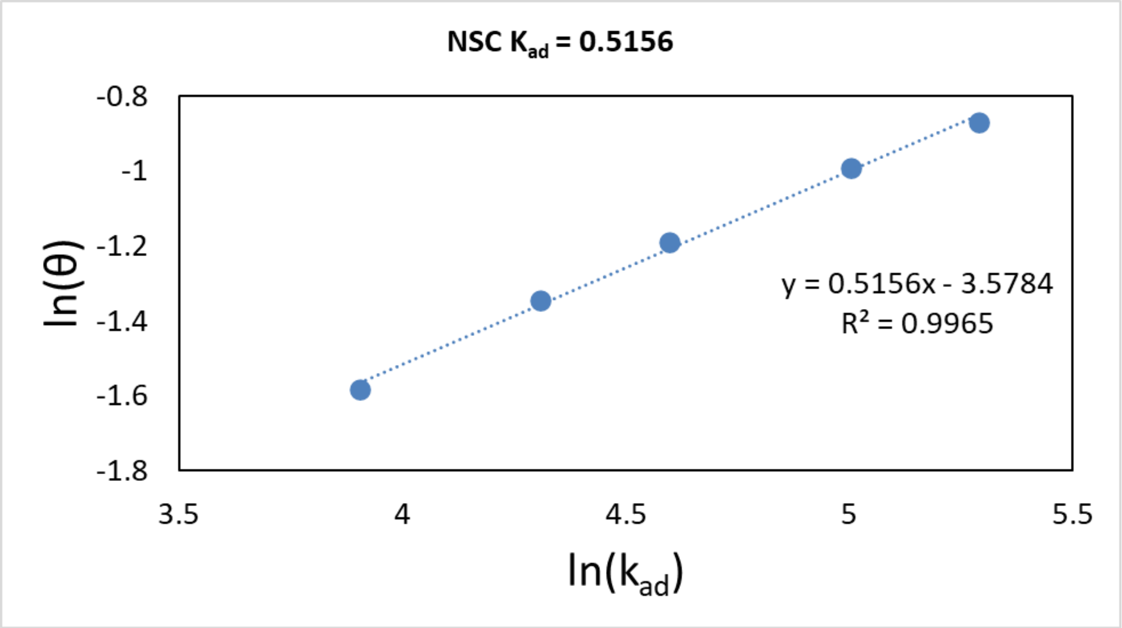

Figure S6: Influence of desorption rate coefficient on the fraction of occupied sites.

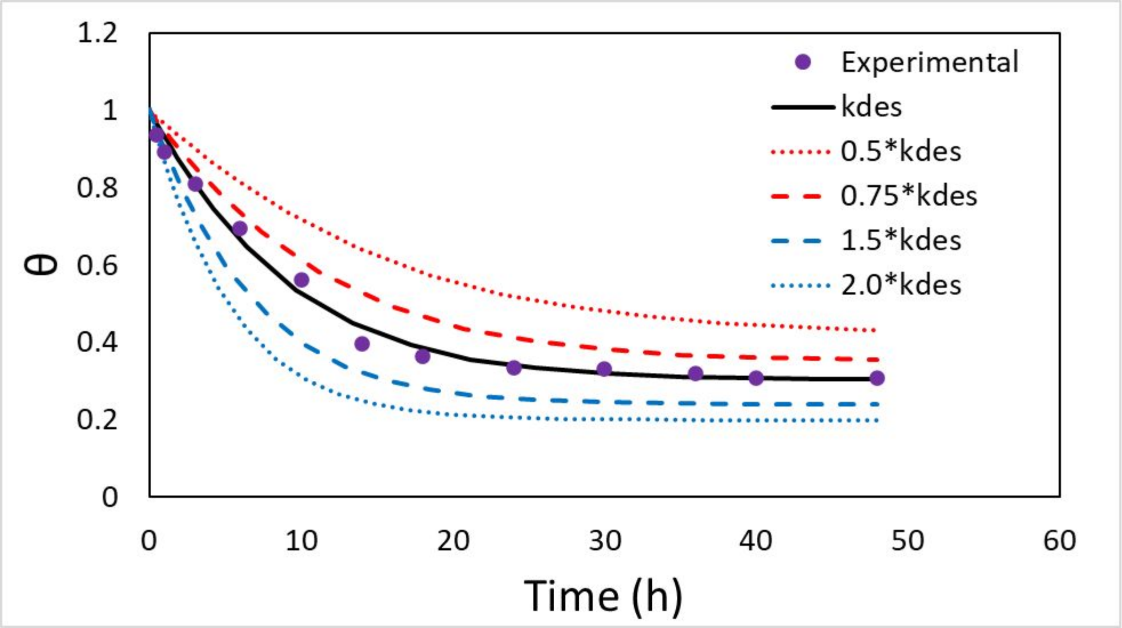

Figure S7: NSC determination for the desorption step

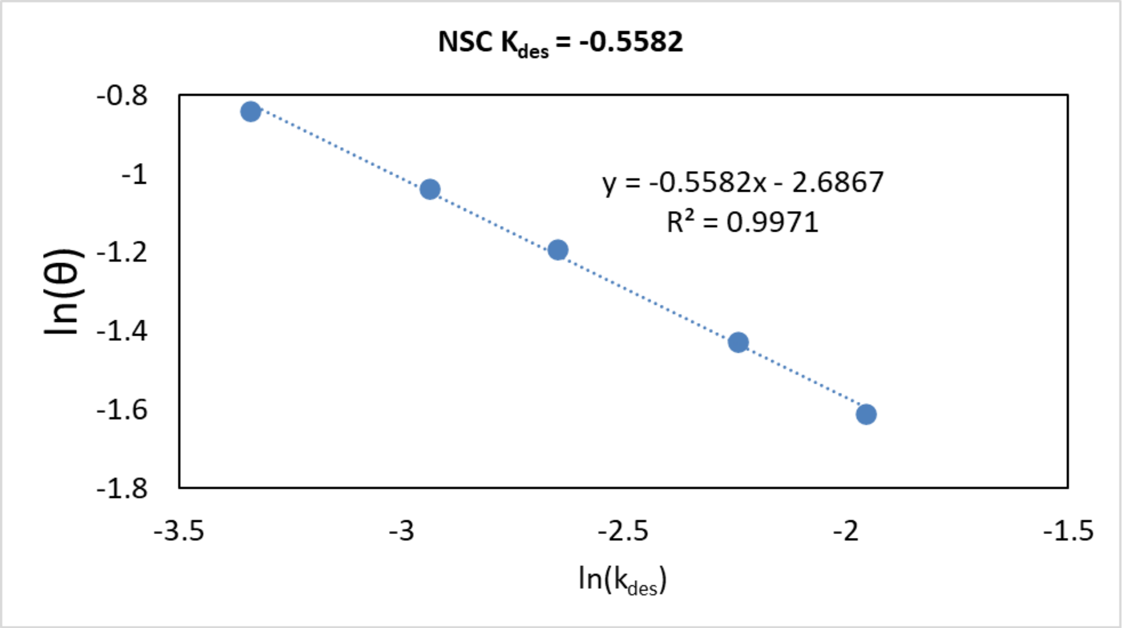

Figure S8: Glyphosate release in aqueous medium from the hybrid compound LDH2-gly as function of the medium's pH. Experimental values are represented by □(PM representing the physical mixing between glyphosate and the hybrid compound),□ (pH 10),□ (pH 8),□ (pH 6) and □ (pH 4). Continuous lines represent fittings according to the two-step kinetic mechanism (TSM), dotted and dashed lines represent pseudo-first-order (P 1st) and pseudo-second-order (P 2nd) kinetic models, respectively.

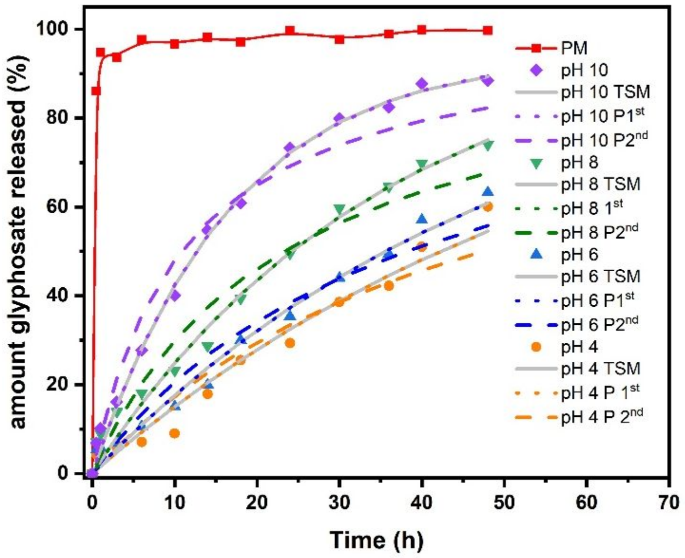

Supplement: Supplementary file 1 [file ao5c10605_si_001.pdf]
